# LINE-1 Methylation sustains telomere length in pregnant women: effects on pregnancy failure

**DOI:** 10.1186/s13148-025-01937-6

**Published:** 2025-07-23

**Authors:** Donato Gemmati, Fabio Scarpellini, Francesca Salvatori, Elisabetta D’Aversa, Roberto Marci, Roberta Capucci, Bianca Antonica, Miriana Grisafi, Elisa Turato, Joanne Vanessa Vargas, Paola Secchiero, Giorgio Zauli, Ajay V. Singh, Veronica Tisato

**Affiliations:** 1 Department of Translational Medicine, University of Ferrara, 44121 Ferrara, Italy; 2 Centre Haemostasis & Thrombosis, University of Ferrara, 44121 Ferrara, Italy; 3 University Strategic Centre for Studies On Gender Medicine, University of Ferrara, 44121 Ferrara, Italy; 4 Centre for Reproductive Medicine, CERM Hungaria, 00193 Rome, Italy; 5 Department of Medical Sciences, University of Ferrara, 44121 Ferrara, Italy; 6 Department of Environmental Sciences and Prevention, University of Ferrara, 44121 Ferrara, Italy; 7 Department of Chemical and Product Safety, German Federal Institute for Risk Assessment (BfR), 10589 Berlin, Germany; 8 LTTA Centre, University of Ferrara, 44121 Ferrara, Italy

**Keywords:** Pregnancy loss, Epigenetics, DNA-methylation, Telomere length, LINE-1, Aging

## Abstract

**Background:**

Pregnancy loss is one of the most common adverse events during the first weeks of gestation, and the incidence increases with maternal age and in presence of selected risk factors. Nonetheless, no risk factors have been identified in most cases, considering these cases unexplained. Fertility rate decreases as maternal age increases and epigenetic age-dependent conditions may favor miscarriage. DNA methylation and telomere length are informative of aging and cell senescence, and their assessment has been evaluated as predictors of successful pregnancy.

**Results:**

Telomere length (TL; T/S) and LINE-1 methylation (LINE-1; %) have been assessed in a cohort of 242 pregnant women by comparing spontaneous early miscarriage (EPL, *n* = 129) with voluntary interruption (VPI, *n* = 113). Telomere size and LINE-1 methylation rate drastically decreased as the age of women increased (*P* < 0.000001) with EPL group having lower values (T/S: 322.6 ± 142.0 *versus* 455.0 ± 290.6; *P* < 0.000001 and LINE-1 %: 81.66 ± 4.2 *versus* 86.01 ± 3.7; *P* < 0.000001) also characterized by stronger age-dependent lowering compared to VPI (*P* = 0.00035 and *P* < 0.000001, respectively). A global improvement in TL was observed as LINE-1 methylation rate increased, and it was more evident in EPL than in VPI (*P* < 0.000001). Focusing on the area below the 25th percentile of TL and LINE-1 % distribution, an overrepresentation of EPL cases was observed (*P* < 0.000001). On the contrary, VPI controls were dramatically overrepresented (*P* < 0.000001) in the area above the respective 75th percentiles. The mutual comparison of the number of EPL and VPI in these two extreme areas (EPL/VPI_(<25th)_ = 3.12 *versus* EPL/VPI_(>75th)_ = 0.32) yielded a significant risk of early pregnancy failure when women carried both risk conditions, low TL and LINE-1 methylation (OR = 9.70, 3.94–23.72; *P* < 0.0001). The intracase analyses ascribed to recurrent EPL cases even higher risks (OR = 10.5, 3.6–29.5; *P* < 0.0001) and a risk dosage effect stratification recognized to low methylation highest odds than that of short telomeres (OR = 4.44, 2.45–8.03; *P* < 0.0001 and OR = 2.26, 1.26–4.04; *P* = 0.005, respectively).

**Conclusions:**

Overall, this suggests a combined effect of short telomeres and low methylation in phenotype worsening and a significant role of methylation in sustaining telomere size. These data support the hypothesis that different levels of DNA methylation may capture different biological mechanisms underlying telomere dynamics and dysfunctions and chromatin organization. Therefore, the concomitant assessment of telomere, methylation and their mutual interactions may be a novel strategy to translate the classical informative biomarkers of aging in the field of human reproduction.

## Introduction

Early pregnancy loss (EPL) is defined as spontaneous pregnancy termination within the 12th gestational week, while recurrent pregnancy loss (RPL) is the failure of two or more consecutive pregnancies before the 20th gestational week [1]. Several risk factors have been associated with both EPL and RPL referring to maternal or fetal conditions, and as for other complex diseases, the genetic/epigenetic mother-fetus crosstalk (GEMCDS) has a great role [2, 3]. Globally, pregnancy loss represents the most common adverse event during the first stages of implantation accounting for a pooled risk of about 15% of all recognized pregnancies [4], while RPL is estimated to be approximately 1 to 2% of couples who are eager for pregnancy [1]. Specifically, the incidence of failure increases with maternal age and in presence of specific risk factors including previous history of miscarriage, chromosomal abnormalities, hormonal and immune unbalancing, endometrium diseases, and inherited or acquired thrombophilia [5–7]. Nonetheless, in about 50% of RPL no recognized risk factors have been identified considering these cases as “unexplained RPL” [8, 9]. Since fertility rate decreases as maternal age increases, aging-dependent factors, by declining genomic stability and the quality and number of oocytes, favor the risk of aneuploidy and in turn of miscarriage [10–12].

DNA methylation and telomere length (TL) are informative of aging and cell senescence in several complex diseases [13, 14] as recently demonstrated in cardiovascular diseases, diabetes, hearing loss, and macular degeneration [15–20]. Preservation of genome integrity is crucial during primordial germ cells (PGCs) and embryo development [21], and assessment of peripheral LINE-1 methylation has been recently evaluated in combination with TL as a predictor of successful in vitro fertilization (IVF) and pregnancy maintenance [22–24]. Several experimental data support a strong link between the activity of DNA methyltransferases (e.g., Dnmt3A and 3B) and an efficient transposon silencing as evidence of methyl-groups deposition at promoters of transposons (e.g., LINE-1) and long-term transcriptional silencing [25, 26]. In experimental embryonic cells, a strict association between maintenance of methylation of repetitive elements and the activity of Dnmt3A and 3B has been provided [27, 28] supporting a coordinated expression of their genes in retrotransposon silencing during oogenesis [29]. This supports that adequate levels of maternal global DNA methylation are crucial for proper oocyte development and survival [30]**.** On the other hand, in early embryos, transposable elements play a role by dynamically re-organizing chromatin arrangement leading to a permissive state revealing a unique spatiotemporal chromatin configuration [31].

Moreover, placental epigenetic alterations by affecting its functions and development may lead to fetal growth restriction, pre-term birth, preeclampsia, and abortion [32]. Although the underlying mechanisms are not fully understood, changes in gene promoter methylation, genomic imprinting, unbalancing in the expression of non-coding RNA (ncRNA), and telomere attrition, are considered the main candidate mechanisms [32]. Hypomethylation-induced genomic instability and telomere shortening may significantly impair placental function by disrupting those cellular processes essential for normal placenta development. The main affected processes, caused by chromosomal abnormalities, such as deletions, duplications, or translocation are those referred to cell division, apoptosis, and gene expression, necessary to support trophoblast invasion and fetal growth [33, 34]. Interestingly, normal term placenta displays marks of senescence including LINE-1 hypomethylation as placenta develops [35], and telomere fusions [36] together with exceptionally high rates of aneuploidy are considered physiological steps in controlling the duration of pregnancy in which telomeres provide the timer [37]. It is to take into account that short placenta TL does not necessarily associate with complicated pregnancy, shortening may be due to LINE-1 hypomethylation and transposon activation caused by TERRA and histone methyltransferase SUV39H1 associated mechanisms [38].

Telomeres are organized by tandem repeats of non-coding DNA sequences (5'-TTAGGG-3') characterized by a terminal region consisting of a single-stranded G-rich 3′-overhang at the end of the chromosomes. Telomeres preserve cell viability since chromosomes naturally shorten with each cell division [39]. As expected, faster telomere shortening occurs in rapidly dividing cells as in cancer [40], and excessive telomere attrition has been observed in various complex diseases such as dementia, autism, diabetes, and hypertension, also associated with oxidative stress or folate isoform unbalancing [3, 41–44]. Variations in TL could be predictive of the specific age at which women reach menopause and in turn the aging rate of oocyte [45, 46]. Few and controversial studies indicate that the relationship between menopause and TL may be dependent on the stage of the menopause, as well as on race and ethnicity, suggesting that additional research should be carried out [47]. In addition, early menarche or menopause, short reproductive lifespan, early age at first birth, multiparity, and use of oral contraceptives or hormone replacement therapy were associated with shorter TL. No significant association was found instead for history of miscarriage and stillbirth [48].

Telomere shortening is counteracted by the enzymatic activity of telomerase, virtually undetectable in most adult somatic cells, and conversely present in cancer cells and adult or embryonic stem and germ cells [49]. On the other hand, the telomerase activity is repressed by TERRA, a long ncRNA Telomeric Repeat-containing RNA, that competitively inhibits the telomerase-telomere binding. Interestingly, TERRA has been recently suggested as predictor of embryo quality and development in assisted reproduction [50, 51]. Moreover, in conditions of physiological or pathological premature aging, LINE-1 hypomethylation and increased nuclear LINE-1 RNA accumulation may induce heterochromatin erosion through an altered H3K9 tri-methylation (i.e., H3K9me3) by the histone methyltransferases SUV39H1 [52]. Accordingly, telomerase defects or its active inhibition associated with shorter telomeres leading to early aging and worsening in oogenesis and egg quality [53].

The complex dynamic of reproduction suggests that telomere shortening in the female germ line is the primary driver of reproductive aging in women. In addition, several studies reported an association between reproductive aging and leukocyte TL, and shorter leukocyte telomeres have been found in unexplained RPL compared to age-matched controls, suggesting a relationship between germ line and somatic telomere size [54, 55]. Finally, an interesting epigenetic model of the connection between pregnancy and aging has been proposed, underlying how the response of pregnant body to gestation may give information on how to counteract the common adverse events as oxidative stress, inflammation, and telomere degradation [56–58]. Considering methylation and telomeres as indicators of reproductive aging in females, in this study we investigated such features in two cohorts of women previously studied for spontaneous pregnancy loss or voluntary pregnancy interruption to disclose their direct role and mutual relationships with the aim to recognize molecular markers of fertility, reproductive aging, and pregnancy outcome.

## Materials and methods

### Study design and samples collection

In the frame of the wider Genetic/Epigenetic Mother–Child Dyad Study (GEMCDS) project [2, 3], aimed at investigating unrevealed mechanisms of the mother-fetus crosstalk responsible for early infancy complex pathological phenotypes, we here approach pregnancy failure being the worst phenotype causing the early death of any living beings. Taking advantage of a previous study assessing the combination of methylation, inherited predispositions and inflammation in pregnancy failure [55], we now analyze a cohort of 242 pregnant women by comparing those who experienced spontaneous miscarriage (EPL, *n* = 129) with a group of pregnant who underwent voluntary interruption according to the Italian law, 194, Art. 6_comma_b (VPI controls, *n* = 113), referring to the Hospital-University of Ferrara, Italy. The study has been approved by the local regional ethical committee (Protocol n. 91–2013, 13/11/2014; PRUA1GR-2013–00000220), and participants provided written informed consent to participate in the study. Exclusion and inclusion criteria are as previously reported [55]. In detail, among the inherited thrombophilic traits, AT, PC, or PS deficiency, or the common gene variants F5 G1691A or F2 G20210A, were considered as non-eligibility for patients to be included in the study.

The EPL group consists of cases with single miscarriage events (sEPL; *n* = 56) and recurrent (rEPL; *n* = 73). We must clarify that women undergoing voluntary pregnancy interruption (VPI) that declared previous spontaneous miscarriage(s) have been excluded from the study, while we could not exclude possible future miscarriages among those women defined as single pregnancy loss (sEPL) at enrollment; ever since a follow-up was not planned in the protocol study. The population characteristics for the whole group and subgroups, EPL-cases and VPI-controls, are summarized in Table 1. Participants had a gestational age ≤ 12 weeks and underwent to whole blood draw in vacutainer containing EDTA or sodium citrate on the day of the programmed intervention, samples were frozen at −80 °C in multiple aliquots and blinded tested.

**Table 1:** Demographic and clinical data of EPL cases and VPI controls

	Whole cohort (n = 242)	EPL cases (n = 129)	VPI controls (n = 113)
age, median (IQR)	34 (28–38)	35 (31–39)	32 (26.5–36)
parity, n (%)			
0	127 (52.5)	92 (71.3)	35 (31.0)
≥ 1	115 (47.5)	37 (28.7)	78 (69.0)
weeks of gestation (mean ± SD)	10.03 ± 1.55	10.5 ± 1.05	9.5 ± 1.8

### Telomere length measurement by Real-Time qPCR

Genomic DNA from each blood sample was extracted using MagCore Genomic DNA Whole Blood kit (RBC Bioscience Corp., New Tampei City, Taiwan) through the automated DNA extraction and purification robot (MagCore Super Automated Nucleic Acid Extractor, RBC Bioscience Corp.) according to the manufacturer’s recommendations. Telomere length was measured using Real-Time qPCR following Cawthon’s method [59], and 36B4 (*RPLP0*) was used as single copy gene reference (SCR). The reaction was performed in a total volume of 25 µl, with SYBR Green PCR Master Mix (Applied Biosystems, California, USA) and 6ng of gDNA. The telomere and 36B4 primer sequences were the same as previously described by Cawthon. Reactions were performed in QuantStudio™3 Real-Time PCR System (Applied Biosystems, California, USA) and all samples were processed in duplicate. Once the Ct (i.e., the number of cycles at which the fluorescence accumulated in the well reaches the pre-established threshold) was determined for each sample, the Telomere/36B4 ratio (T/S) was assessed by calculating 2^−∆Ct^ (△Ct = Ct telomere − Ct 36B4) as previously described [59]. The T/S ratio is one of the most widely used methods for measuring TL, especially in large-scale epidemiological studies. Thanks to its scalability, it can be applied in a high-throughput format. Possible variations between qPCR plates have been verified by a standardized reference pool sample of DNA isolated from a group of women with matched age-range included in every plate/run. The validity of the analysis was contingent upon the consistent Ct values obtained from this internal reference across all plates.

### LINE-1 methylation by pyrosequencing

Genomic DNA from each blood sample was extracted using MagCore Genomic DNA Whole Blood kit (RBC Bioscience Corp.) through the automated DNA extraction and purification robot (MagCore Super Automated Nucleic Acid Extractor, RBC Bioscience Corp.) and 500 ng were bisulfite-converted by using EpiTect Bisulphite kit (Qiagen, Hilden, Germany) in a final volume of 70µl in accordance with the manufacturer’s recommendations. Converted DNA (20µl) was amplified via a bisulfite-sensitive PCR assay using the Pyromark PCR kit (Qiagen, Hilden, Germany) and LINE-1 specific primers (Fw: 5′-TTTTGAGTTAGGTGTGGGATATA-3′; Rev: 5′Bio-AAAATCAAAAAATTCCCTTTC-3′) in a final volume of 50µl. The reaction was performed on the SureCycler_8800 (Agilent Technologies, Mulgrave, AU) according to the following protocol: an initial step at 95 °C for 15 min; followed by 40 cycles at 94 °C for 30 s; 50 °C for 30 s; 72 °C for 30 s; and a final extension at 72 °C for 10 min. The specificity of the LINE-1 PCR was verified by 1.8% agarose gel electrophoresis to distinguish the 146 bp sequence amplified by the reaction. The sample pyrosequencing was performed on a PyroMark Q48 (Qiagen, Hilden, Germany) using the LINE-1 sequencing primer (5′-AGTTAGTGTGGGATATAGT-3′) and PyroMark Q48 Advance Reagents (Qiagen). The methylation of CpG dinucleotides was calculated as the percentage of cytosine nucleotides relative to the sum of cytosine and thymine nucleotides at a given location by Pyromark Q48 v2.4.2 software. The overall % of LINE-1 DNA methylation level was calculated as previously reported [15, 55]. To ensure accuracy and reproducibility of LINE-1 methylation analysis, internal controls were employed in the experimental workflow. Specifically, the three commercially available quality-controlled human DNA samples (EpiTect® PCR Control DNA Set, Qiagen, Hilden, Germany) were included and processed in each analysis. The methylated and unmethylated bisulfite-converted DNAs were used to verify primer specificity for bisulfite-converted DNA and efficiency when performing methylation-specific end-point PCR. Finally, unmethylated genomic DNA was used to test efficiency of bisulfite conversion. Pyrosequencing for DNA methylation analysis is supported by its quantitative nature and CpG-site specificity offering an easy cost-effective toll of analysis.

### Statistical analyses

Data recording and statistical analyses have been performed by using SPSS Statistics version 22.0 (SPSS, Inc., Chicago, IL, USA) and MedCalc version 20.112 (MedCalc Software Ltd., Ostend, Belgium). Statistical significance between clinical parameters of EPL cases and VPI controls has been determined by Chi-Square test and Student’s t-test. Normal distribution of LINE-1 and telomere size distributions have been tested by Kolmogorov–Smirnov test and the significance of changes calculated by Mann–Whitney U test. To estimate associations between LINE-1 methylation and telomere size (or combinations) with the risk of pregnancy loss, odds ratio (OR) and 95% confidence interval (CI) have been used. Spearman’s rank correlation coefficient test has been used to estimate relationships between LINE-1 methylation, telomere length, age, and mutual combinations. All figures were produced by GraphPad Prism9 (GraphPad Software, Inc., San Diego, California USA) or MedCalc version 20.112 (MedCalc Software Ltd., Ostend, Belgium) unless otherwise specified. Telomere T/S ratio and methylation % have been centered and scaled to become normalized before correlation sub-analyses according to the formula (*x*-value—mean value)/SD; [Z = (x—μ)/σ]. Data were expressed as mean ± standard deviation (SD) or median and interquartile ranges (IQR). Two-tailed probability and P values less than 0.05 have been considered statistically significant.

## Results

### Telomere length and age

**Fig. 1:**
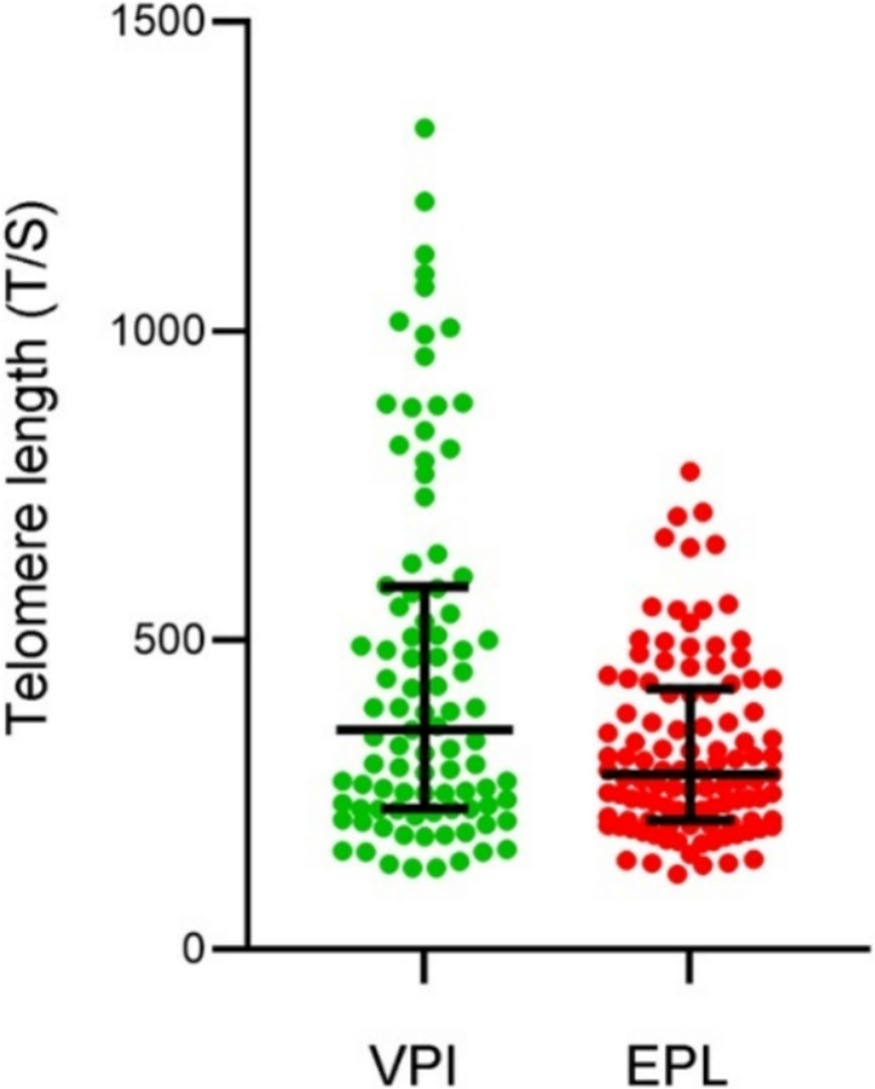
Telomere length distribution assessed as T/S ratio in VPI controls (green dots) and EPL cases (red dots). Black lines indicate median and IQR

The main characteristics of the population investigated are shown in Table 1. Among the 242 cases, 202 (83.5%) had available samples to assess telomere length. Figure 1 shows the telomere length distribution in the EPL (*n* = 109; 53.9%) and VPI (*n* = 93; 46.1%) subgroups expressed as T/S ratio. Both T/S mean (322.6 ± 142.0 *versus* 455.0 ± 290.6) and median T/S (283.0 *versus* 354.6) were significantly lower in the EPL compared to VPI subgroup (*P* < 0.000001). The higher mean age in the EPL subgroup compared to VPI did not change the statistical significance when the telomere length measurement was corrected for age (adjusted *P* value < 0.00001).

**Fig. 2:**
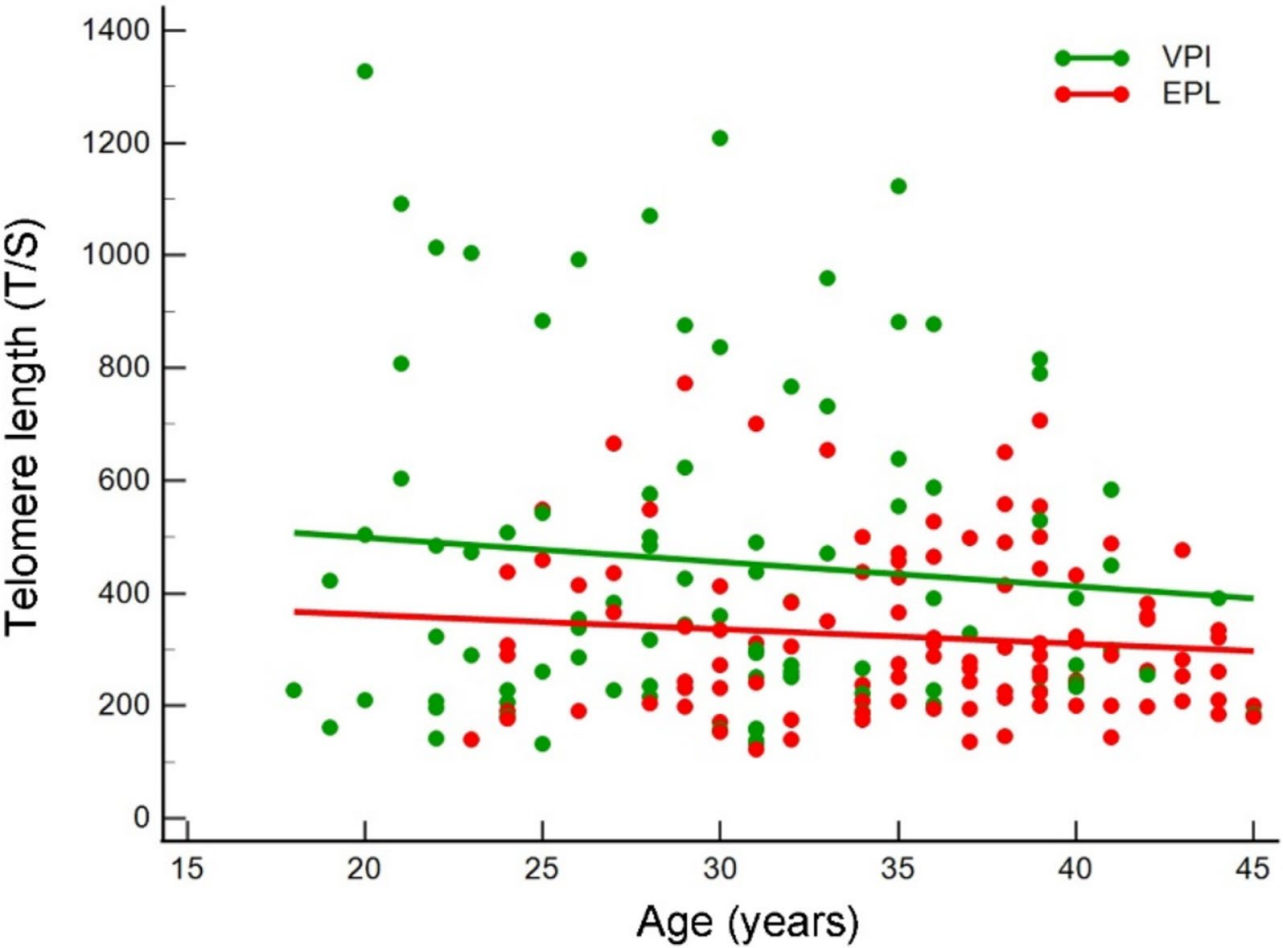
Telomere length distribution assessed as T/S ratio stratified by age in EPL cases (red dots) and VPI controls (green dots). Regression lines are shown (green line and red line for VPI controls and EPL cases respectively)

Furthermore, considering the age dependence of telomere length, we stratified EPL cases and VPI controls by age. As shown in Fig. 2, EPL subgroup had a significantly lower T/S value at any age considered compared to VPI subgroup. The two regression lines yielded significantly different intercept coefficients (*c*_*i*_ = 413.78 and *c*_*i*_ = 585.57, respectively; *P* = 0.00035) and comparable slopes (*r*^*2*^ = 0.0107 and *r*^*2*^ = 0.0099 respectively; *P* = 0.734). This was mainly due to the appreciable presence of shorter telomeres in younger cases in both subgroups. As for the T/S mean comparison (see above), the regression lines kept significance also by comparing the two age-matched subgroups (adjusted *P* = 0.003).

### LINE-1 methylation and age

**Fig. 3:**
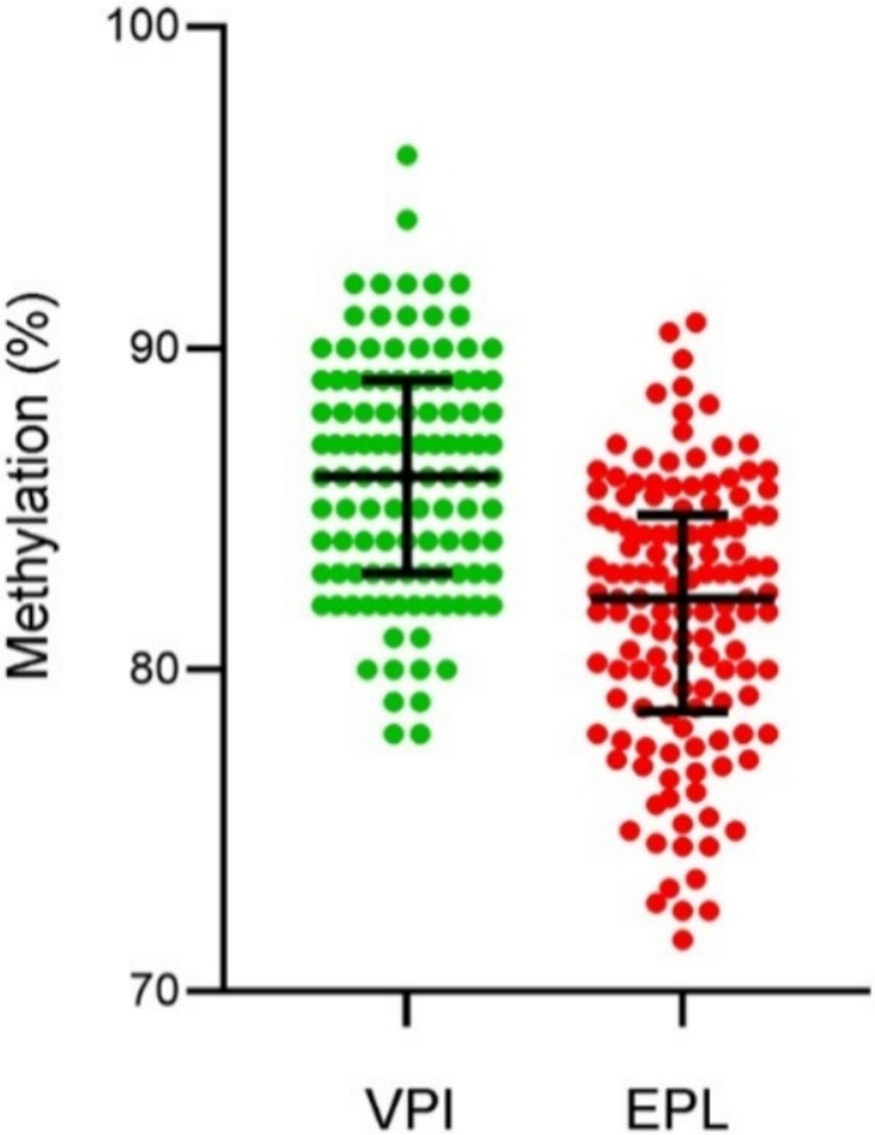
LINE-1 methylation distribution assessed as a percentage (%) in VPI controls (green dots) and EPL cases (red dots). Black lines indicate median and IQR

Globally, all the enrolled cases (*n* = 242) had available samples to assess methylation levels. Figure 3 shows LINE-1 methylation in EPL (*n* = 129; 53.3%) and VPI (*n* = 113; 46.7%) subgroups expressed as the mean of the cytosine percentage of the CpG sites analyzed. Mean LINE-1 methylation (81.66 ± 4.2 *versus* 86.01 ± 3.7) and median (82.2 *versus* 86.0) were significantly lower in the EPL compared to VPI subgroup (*P* < 0.000001). The higher mean age in the EPL subgroup compared to VPI did not change the statistical significance when the test was corrected for age (adjusted *P* value < 0.00001).

**Fig. 4:**
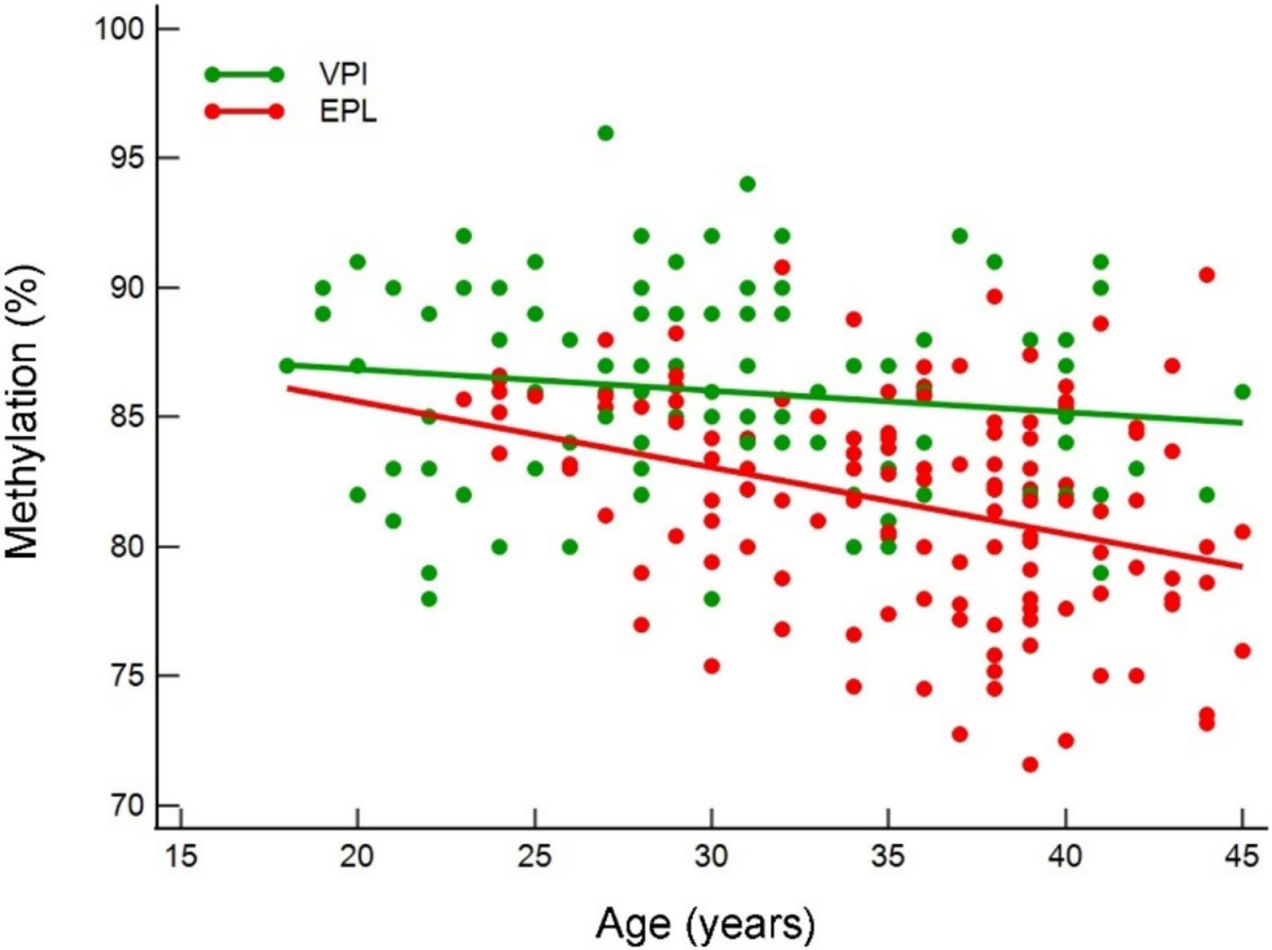
LINE-1 methylation distribution assessed as percentage (%) stratified by age in EPL cases (red dots) and VPI controls (green dots). Regression lines are shown (green line and red line for VPI controls and EPL cases respectively)

Given that age affects the methylation status, we stratified EPL cases and VPI controls by age. Figure 4 shows that EPL had a significantly stronger lowering in LINE-1 methylation as age increases compared to VPI (*r*^*2*^ = 0.113 *versus r*^*2*^ = 0.0217; *P* = 0.03) showing appreciable different regression lines (*c*_*i*_ = 90.7 *versus c*_*i*_ = 88.5; *P* < 0.000001), and the regressions kept significant difference also by comparing the age-matched subgroups (*P* = 0.00001).

### Correlation between telomere length and LINE-1 methylation

**Fig. 5:**
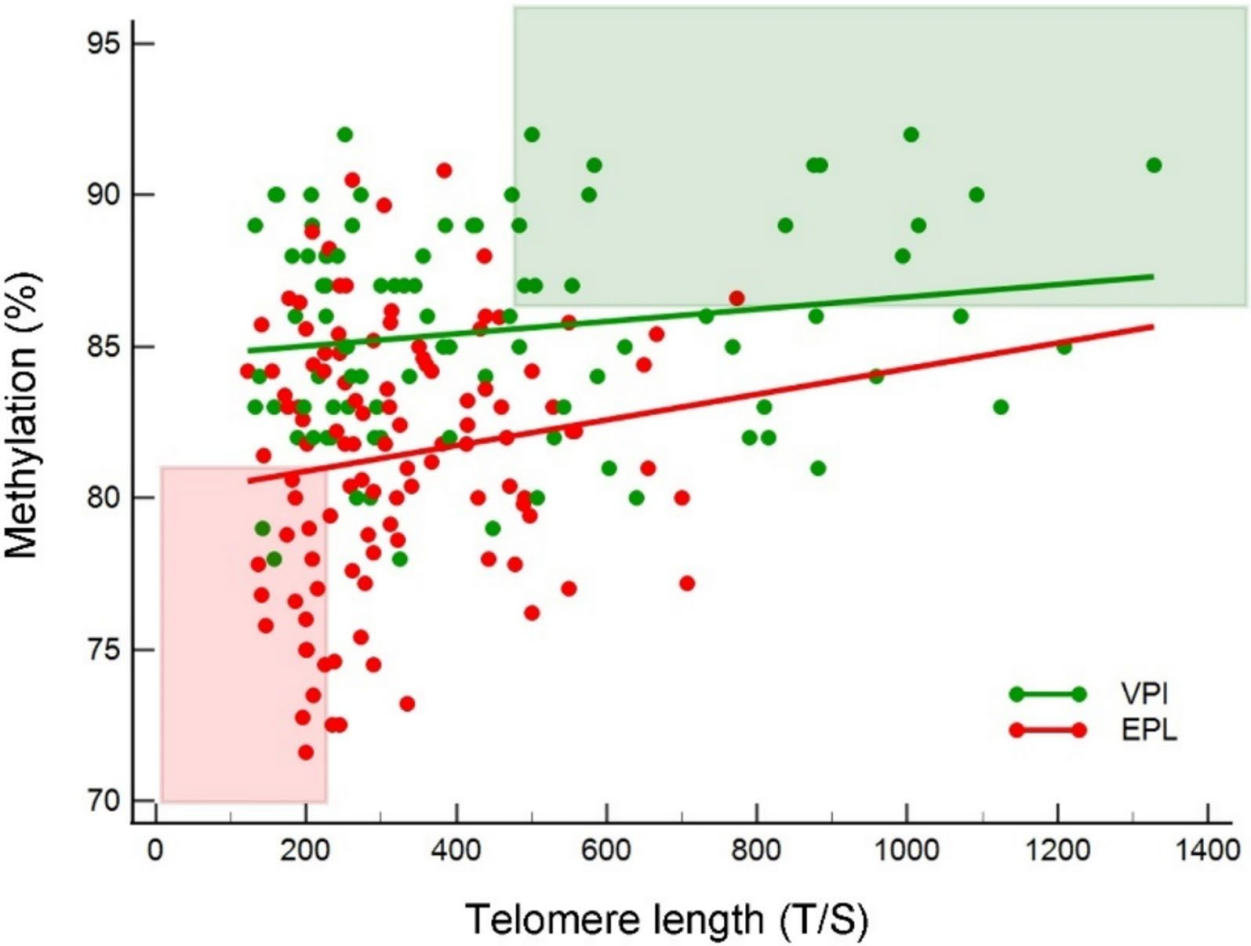
LINE-1 methylation (%) distribution stratified by telomere length (T/S) distribution in EPL cases (red dots) and VPI controls (green dots). Regression lines are shown (green line and red line for VPI controls and EPL cases, respectively). The red box (bottom left) represents those EPL cases and VPI controls having both LINE-1 methylation (%) and telomere length (T/S) below the 25th percentile of the respective global distributions. The green box (upper right) represents those EPL cases and VPI controls having both LINE-1 methylation (%) and telomere length (T/S) above the 75th percentile of the respective global distributions

As further investigations, provided that age negatively affects both telomere length and methylation percentage, we investigated their mutual interaction in the two subgroups. Figure 5 shows a global improvement in telomere length as methylation level increases, strongly evident in both EPL and VPI subgroups, showing regression lines with similar slopes (*r*^*2*^ = 0.0203 and *r*^*2*^ = 0.0282, respectively; *P* = 0.459). On the other hand, VPI subgroup showed persistent higher mean methylation levels throughout telomere length distribution than EPL cases with higher different intercept coefficients (*c*_*i*_ = 80.04 *versus c*_*i*_ = 84.61; *P* < 0.000001). Interestingly, focusing on the area below the 25th percentile of the distribution of the two variables (i.e., methylation ≤ 81.0% and telomere length T/S ≤ 225.0), a significant overrepresentation of EPL cases was observed (89.5%) (Fig. 5, red box). On the contrary, a significant overrepresentation of VPI controls (94%) was observed above the respective 75th percentile (i.e., methylation ≥ 86.6% and telomere length T/S ≥ 476.5) (Fig. 5, green box).

**Fig. 6:**
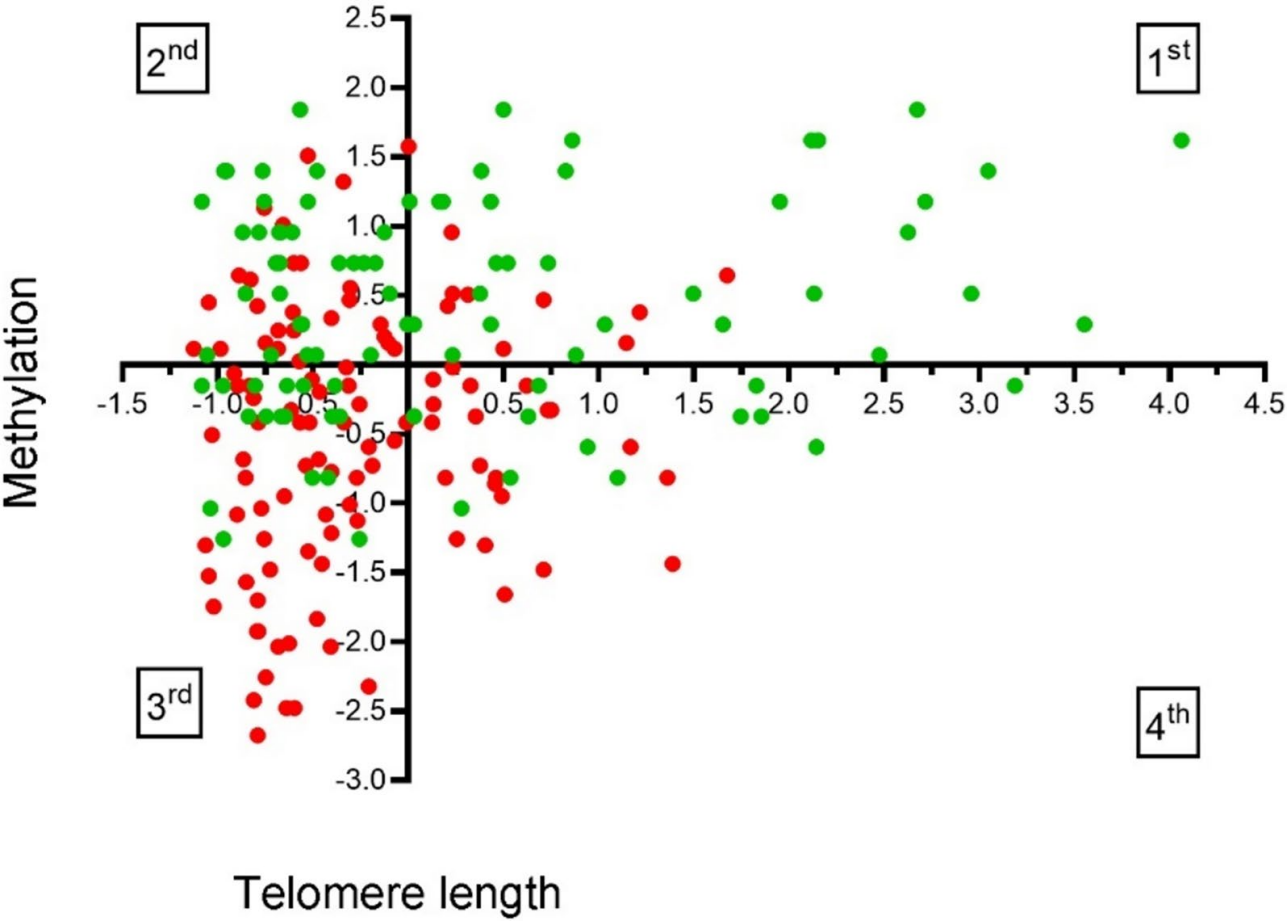
Normalized LINE-1 methylation distribution stratified by normalized telomere length distribution in EPL cases (red dots) and VPI controls (green dots) as described in the Methods section. Cases and controls within the first quadrant have LINE-1 methylation and telomere values both greater than the normalized mean values; those within the third quadrant have values both below the normalized mean values; those cases and controls within the second and fourth quadrants have, respectively, alternative telomere or LINE-1 methylation below the normalized mean values and vice versa

To disclose if a specific cut-off of methylation might sustain different telomere sizes, we stratified both EPL and VPI cases by normalized telomere and methylation values as detailed in the Methods section. As a result, Fig. 6 was divided into four different quadrants stratifying the normalized T/S ratio (X-axis) and the normalized methylation percentage (Y-axis) as detailed below: X- and Y-values both greater (positive) than the normalized mean values (1st upper right corner); X- and Y-values both lower (negative) than the normalized means (3rd bottom left corner); and alternatively X-negative and Y-positive (2nd upper left corner) and X-positive and Y-negative (4th bottom right corner). Interestingly, the rate of spontaneous EPL was maxima (EPL/VPI = 3.12) when both T/S and methylation were in the low ranges (3rd bottom left corner) and minima (EPL/VPI = 0.32) when both T/S and methylation were above the mean values (1st upper right corner) with a differential gap of about 10-folds (*P* < 0.0001). The remaining two quadrants (i.e., 2nd upper left and 4th bottom right corner) showed intermediate rates (EPL/VPI = 0.78 and 1.62 respectively) with a differential gap of about 2.0-folds still not significant (*P* = 0.130).

### Pregnancy loss risk assessment

The higher risk of spontaneous abortion was attributed to those cases who presented both risk conditions, which consists of telomere length and methylation rate below the normalized mean values (i.e., 3rd quadrant of the distribution), compared to cases with telomere size and methylation rate above normalized mean values (i.e., 1st quadrant), accounting for an OR = 9.70 (C.I. 95%, 3.94–23.72; *P* < 0.0001). Considering separately only one of the two risk conditions, the OR calculation ascribed to low methylation rate a higher risk value than that of short telomere length alone, being anyhow both appreciable and significant (OR_LowMeth_ 4.44, C.I. 95%, 2.45–8.03; *P* < 0.0001) and (OR_ShortTL_ 2.26, C.I. 95%, 1.26–4.04; *P* = 0.005). According to the crude OR-values, we can advise for an additive effect for the coexistence of the two risk conditions as follows: low methylation plus short TL >  > low-methylation and normal TL > short TL and normal methylation.

### Intracase correlation analyses (sEPL versus rEPL)

**Fig. 7:**
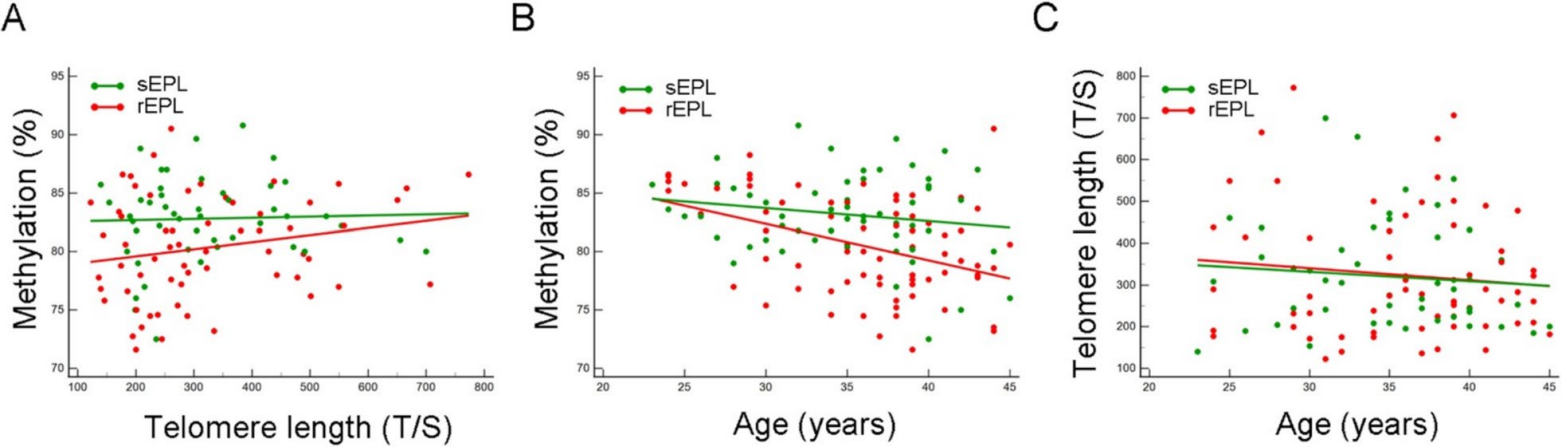
LINE-1 (%) distribution stratified by telomere length (T/S ratio) and age distribution (**A** and **B,** respectively), and telomere length distribution (T/S ratio) stratified by age (**C**) in recurrent EPL cases (rEPL, red dots) and single EPL (sEPL, green dots)

To further investigate the combined involvement of telomere size and methylation rate in pregnancy maintenance, we also stratified cases by single EPL event (sEPL; *n* = 56) and recurrent EPL (rEPL; *n* = 73). Interestingly, a direct correlation between methylation and telomere length was stronger in rEPL than in sEPL (*c*_*i*_ = 78.38 *versus c*_*i*_ = 82.49; P = 0.002) keeping slopes (r^2^ = 0.045 *versus* r^2^ = 0.001; *P* = 0.378) (Fig. 6A) suggestive of the associated additive risk effect. Instead, aging seems to strongly affect methylation in rEPL compared to sEPL (*c*_*i*_ = 91.6 *versus c*_*i*_ = 87.07; *P* = 0.0005) and (r^2^ = 0.167 *versus* r^2^ = 0.028; *P* = 0.101) (Fig. 6B), while telomeres attrition was similarly affected by age in the two subgroups (Fig. 6C). Overall, this suggests, on the one hand, the combined effect of telomeres and methylation in the worsening of phenotype, and on the other hand a significant role of methylation in sustaining telomere length. As a result, rEPL shared a not significantly higher risk of failing a pregnancy than sEPL cases (OR = 1.20; C.I. 95%, 0.3–4.8; *P* = NS) but an increased risk of recurrence when compared to VPI controls (OR = 10.5; C.I. 95%, 3.6–29.5; *P* < 0.0001). As for the whole group, the single risk factor analysis ascribed to low methylation higher risks than short telomeres in both subgroups (OR_rEPL_ = 5.5; C.I. 95%, 2.75–10.99 and OR_sEPL_ = 3.33; C.I. 95%, 1.6–7.03) and (OR_rEPL_ = 2.2; C.I. 95%, 1.12–4.26 and OR_sEPL_ = 2.3; C.I. 95%, 1.09–5.16) for low methylation and low telomere size, respectively (Fig. 7).

## Discussion

The role of appropriate telomere length and genome methylation in human reproduction is now widely recognized [23, 55, 60–62]. The relationships between epigenetics and telomere dynamics have been extensively investigated but remain partly unknown mainly because several interfering factors such as inflammation, oxidative stress, and concomitant diseases may affect any conclusive result [62]. In particular, numerous studies investigated the direct relationship between LINE-1 methylation and TL [63–66], disclosing evidence for negative or positive associations in several complex diseases such as diabetes, metabolic syndrome, cancer, and intrauterine growth restriction [65, 67, 68]. The contradictory associations might also relate to the different diseases, ethnicity, use of vitamin supplements, or different methodology applied [64, 69, 70]. Instead, telomere dynamics have also a strong impact on DNA methylation by regulating cell senescence and chromosome stability. For instance, the accumulation of DNA damage due to aging, chronic inflammation, and oxidative stress, by deregulating telomere homeostasis and integrity may affect in turn the DNA methylation balance [71, 72]. Summarizing, constitutive heterochromatin is responsible for genome repression of DNA enriched of repetitive sequences as in telomeres and centromeres. Accordingly, lower global DNA methylation is associated with shorter TL in several pathological conditions, as early pregnancy loss. The reason is that, whereas DNA methylation is one of the mechanisms that control TL and its maintenance, telomere dysfunction can cause LINE-1 retrotransposition, activation, and genome instability disrupting those host genes strongly involved in early embryo development [65]. Interestingly, this concept has been recently expanded and taken up studying the similarities between pregnancy and aging pathologies [56–58].

In the present paper, we assessed LINE-1 methylation and TL in two groups of pregnant women characterized by spontaneous miscarriage (EPL) or voluntary pregnancy interruption (VPI) to disclose mutual relationships and the underlying mechanisms. Both groups showed a significant direct decrease in telomere size and methylation rate as age of women increased and the EPL group had significant lower mean levels and stronger age-dependent lowering compared to VPI. These data might ascribe to EPL cases a potential higher accumulation of global genome damage (constitutive and/or acquired) during their life or in the periconceptional period than that physiologically presumed in normal healthy women with comparable age, ethnicity and coming from the same geographical region (i.e. VPI controls).

Although at different extents, age negatively affects both telomere size and methylation accounting for a gap of more than 10 years in the two groups, with EPL cases constantly characterized by lower mean findings. This has been further confirmed in the intracase analysis, suggesting a basic dysregulation of the epigenetic mechanisms essential for the maintenance of a healthy pregnancy [22, 73]. In this line, correlations between epigenetic clocks and anti-Müllerian hormone, or ovarian reserve, or successful IVF have been reported [22, 74, 75], proposing that accelerated epigenetic mechanisms might determine pregnancy outcome [76] and that epigenetic clocks, by measuring age acceleration via DNA methylation and telomere size, may associate with decreased oocyte yield [55, 74].

Reproductive lifespan and fertility in females drastically decrease as ovarian age advances due to the worsening of oocyte reserve and quality [77–79]. Moreover, biological aging also affects endometrium receptivity and placenta development making successful pregnancy challenging also after IVF procedures [80, 81]. Accordingly, LINE-1 hypomethylation and LINE-1 RNA temporal regulation appeared to be specific epigenetic entities in placenta development, suggesting a link between LINE-1 activation and proliferation of specific placenta cellular subsets [34]. In this line, fetal growth restriction and preeclampsia have been associated with accelerated telomere shortening, and increased expression of placenta senescence markers [82, 83] and early placental aging were linked with aberrant changes in TL, cellular senescence, and mitochondrial dysfunction [84, 85]. However, previous investigations reported conflicting findings, stating that placental TL was not related to severe preeclampsia but negatively associated with gestational age [86], supporting the hypothesis that telomere shortening at term contributes to the mechanism that determine the length of pregnancy thereby leading to onset of parturition [87].

Telomere attrition and DNA methylation are related to biological aging and these intricate mechanisms have been proposed and confirmed in several complex diseases, including failure of pregnancy maintenance [13–15, 55, 88, 89]. In detail, hypomethylation of retrotransposable DNA elements (e.g., LINE-1 or Alu) and subtelomeric regions in peripheral blood leukocytes have been associated with shorter telomeres and elevated oxidative DNA damage. Then, considering these regions important in telomere maintenance, finding out possible links between global DNA methylation and telomere size preservation may serve as potential biomarker identification [88, 90, 91].

For these reasons, to disclose their mutual interactions and to identify which combination was most strongly associated with pregnancy loss, we stratified EPL cases and VPI controls by normalized telomere size and methylation levels revealing a pooled risk dosage-effect [6, 7] on the risk of pregnancy failure. Women combining telomere and methylation both below the average values had the highest risk (i.e., 9.70-folds), compared to those with low methylation (i.e., 4.44-folds) or short telomere (i.e., 2.26-folds). Moreover, the intracase analysis straightened similar information assigning to the recurrent miscarriage subgroup even slightly higher risks (OR = 10.5-folds) and stronger telomere size-methylation correlations sharing, on the contrary, overlapping trends for the age-dependent telomere shortening.

These parameters are of high importance in clinical practice because of the growing concern about ovarian aging and both male and female fertility issues, that are forcing couples to resort to assisted reproduction technologies (ART) to achieve a successful pregnancy [92, 93]. While telomere size is related to life expectancy and reproductive lifespan [61, 94], immature oocytes have longer telomeres (11.41 kb) than mature oocytes (8.79 kb) in humans [95, 96], and the shortening during oocyte maturation is not due to DNA replication because oocytes are arrested in prophase I. The exposure to an aged microenvironment may result in telomere shortening, as shown in the ovaries of older women and mice [97, 98]. Consequently, oocyte competence reduces with age due to unrepaired accumulated DNA damage and different meiotic abnormalities [61, 99, 100]. Optimal methylation may protect and preserve DNA erosion from damage and instability, particularly in telomeric and subtelomeric regions, these latter enriched in CpG and LINE-1 elements. Moreover, DNA hypomethylation could affect the D-loop-T-loop structure at the telomere-end and disturb the binding of telomere-associated proteins useful to eliminate DNA free-ends that may cause telomeric dysfunctions as double- or single-stranded DNA breaks [89].

In particular, when subtelomeres are abnormally hypomethylated, subtelomeric heterochromatin shifts into an open structure favoring TERRA (TElomeric Repeat-containing RNA) high expression to inhibit telomere lengthening by telomerase enzyme, causing in turn rapid telomere erosion. Accordingly, prolonged DNA hypomethylation not only leads to excessive telomere shortening but also to telomere truncation, accelerating in turn cell aging [91, 101, 102]. In the field of human reproduction, in a pilot study recruiting normo-zoospermic (NZ) and oligo-asthenozoospermic (OAZ) males attending the ART procedure, OAZ had altered telomere maintenance, shorter TL in spermatozoa, and lower quality embryos compared to NZ samples. Although, NZ and OAZ groups had similar levels of TERRA intensity in spermatozoa, the number of TERRA foci increased in OAZ samples, suggesting TERRA molecules grouped differently compared to controls [103]. Similarly, it is the role of the epigenetic reprogramming gene SUV39H1 histone methyltransferase in preserving constitutive heterochromatin, suppressing DNA accessibility and favoring gene silencing [104] being significantly altered in cryopreserved oocytes when compared to controls [38].

Although we did not assess telomere size and methylation in female gametes or uterus, several studies have explored the alternative of measuring telomeres in other cell types to find an indirect marker of oocyte telomere parameters. The cell types proposed are polar bodies [105], leukocytes [106], and follicular cells [107]. In detail, leukocytes have also been considered as a possible indicator of oocyte telomere status considering that the onset of menopause in women with long telomeres in blood begins later compared to those with short telomeres [45]. In turn, by comparing women of the same age with and without menopause, women in the postmenopausal period had shorter telomeres [45]. Genome methylation is the primary epigenetic silencing mechanism of endogenous transposons, imprinted genes, and pluripotency-related genes in somatic cells, and is one of the commonest tool used in epigenetic mapping [108–110]. By using a DNA methylation estimator of TL (DNAmTL) it was possible to better assess and predict several age-related diseases as age-to-menopause. This is because DNAmTL dynamics also reflect cell replication rather than merely telomere size [111].

Accordingly, longer telomeres in leukocytes were associated with higher fertility and better oocyte quality, which can compensate for other factors with a negative effect on reproduction [112]. Similarly, LINE-1 methylation from whole blood cells is a recognized surrogate of global genome methylation and aging, as reported in several previous studies on general health status, complex diseases, and particularly in pregnancy loss and association with decreased oocyte yield in women [15, 55, 113, 114]. Globally, maternal epigenetics condition may influence endometrium receptivity, crucial for successful embryo implantation and pregnancy outcome.

Summarizing, epigenetics modifications during gestation can drive a range of molecular changes that contribute to pathological conditions, and pregnancy itself can impact on maternal DNA methylation and telomere length [57]. This loop has been recently proposed suggesting that pregnancy offers an exceptional platform for examining stress and stress responses demonstrating accelerated epigenetic aging in women with complicated gestation [57, 115, 116]. Finally, the strategy we applied in the present research is the concomitant assessment and mutual interaction between methylation score and telomere size. This action is individually experienced by each pregnant woman and gives novel personalized and global readings of the basal epigenetic status of the individual response and on how coping environmental and endogenous conditions.

## Conclusions

The present data support the hypothesis that different levels of DNA methylation may capture different biological mechanisms underlying telomere dynamics and dysfunctions. Women experiencing pregnancy loss may less efficiently respond (e.g., epigenetically) to the exogenous or endogenous stressors of the perigestational period. Therefore, the concomitant assessment of telomere size, methylation rate and their mutual interactions may be a strategy to translate the classical informative biomarkers of aging in the field of human reproduction. Identifying predictive indicators of pregnancy outcome may also embrace the recognizing of novel indirect estimators of reproductive lifespan and fertility and have the potential to become applicable tools for successful embryo implantation after in vitro fertilization paving the way for new approaches in reproductive medicine [117].

## Data Availability

The data related to this article are available within the article.
